# MaioRegen Osteochondral Substitute for the Treatment of Knee Defects: A Systematic Review of the Literature

**DOI:** 10.3390/jcm8060783

**Published:** 2019-06-01

**Authors:** Riccardo D’Ambrosi, Federico Valli, Paola De Luca, Nicola Ursino, Federico Giuseppe Usuelli

**Affiliations:** 1 IRCCS Istituto Ortopedico Galeazzi, CASCO Department, 20161 Milan, Italy; mail@federicovalli.it (F.V.); nicolaursino@libero.it (N.U.); fusuelli@gmail.com (F.G.U.); 2 IRCCS Istituto Ortopedico Galeazzi, Orthopaedic Biotechnology Lab, 20161 Milan, Italy; deluca.paola@grupposandonato.it

**Keywords:** osteochondral defect, scaffold, cartilage, graft, MaioRegen, osteochondral substitute

## Abstract

Background: This study aims to investigate the clinical and radiological efficacy of three-dimensional acellular scaffolds (MaioRegen) in restoring osteochondral knee defects. Methods: MEDLINE, Scopus, CINAHL, Embase, and Cochrane Databases were searched for articles in which patients were treated with MaioRegen for osteochondral knee defects. Results: A total of 471 patients were included in the study (mean age 34.07 ± 5.28 years). The treatment involved 500 lesions divided as follows: 202 (40.4%) medial femoral condyles, 107 (21.4%) lateral femoral condyles, 28 (5.6%) tibial plateaus, 46 (9.2%) trochleas, 74 (14.8%) patellas, and 43 (8.6%) unspecified femoral condyles. Mean lesion size was 3.6 ± 0.85 cm^2^. Only four studies reported a follow-up longer than 24 months. Significant clinical improvement has been reported in almost all studies with further improvement up to 5 years after surgery. A total of 59 complications were reported of which 52 (11.1%) experienced minor complications and 7 (1.48%) major complications. A total of 16 (3.39%) failures were reported. Conclusion: This systematic review describes the current available evidence for the treatment of osteochondral knee defects with MaioRegen Osteochondral substitute reporting promising satisfactory and reliable results at mid-term follow-up. A low rate of complications and failure was reported, confirming the safety of this scaffold. Considering the low level of evidence of the study included in the review, this data does not support the superiority of the Maioregen in terms of clinical improvement at follow-up compared to conservative treatment or other cartilage techniques.

## 1. Introduction

Achieving a predictable and durable repair after an articular cartilage knee injury is still a clinical challenge for orthopedic surgeons. In recent years, advancements in arthroscopy and imaging have led to an increase in the acute recognition of chondral and osteochondral defects [1,2,3]. Curl et al. reported a percentage of 63% of patients with chondral injury considering 31,516 knee arthroscopies; this condition affects approximately 900,000 Americans annually resulting in more than 200,000 surgical procedures [4,5]. Cartilage injuries can result in pain, swelling, clicking, instability, and finally progression to a more diffuse degenerative process [6,7]. Current surgical treatment includes arthroscopic debridement and arthroscopic bone marrow stimulation including microfractures, used either alone or in combination with scaffold such as in the case of the autologous matrix-induced chondrogenesis (AMIC) technique that combines microfractures with a collagen I/III membrane [8]. Other treatments include autologous or allogenic osteochondral transplantation as well as natural or synthetic scaffolds used alone or in combination with cells. Finally, cell-based therapies, such as autologous chondrocyte implantation (ACI) represent an effective, although expensive clinical treatment for patients affected by focal chondral lesions. More recently, second-generation methods to improve the outcome of the ACI procedure using three-dimensional scaffolds have been described (MACI, matrix-induced ACI) [9,10,11].

Although the use of allogenic osteochondral grafts might appear a good strategy, the complex biological events behind allograft integration as well as the lack of availability of allografts in many countries limit their use [12]. To overcome these drawbacks, the last few years have seen the development of new bioengineered scaffolds for the treatment of cartilage defects [10]. When implanting an osteochondral scaffold at the lesion site, the aim is to promote tissue repair through the formation of a tissue that is as similar as possible to the native one, with physiological properties similar to those of the entire osteochondral unit, and therefore durable over time [11]. In recent years new biomimetic three-dimensional acellular scaffolds (MaioRegen; Fin-Ceramica Faenza SpA) have been proposed for “in situ” cartilage regeneration [13]. MaioRegen is a nanostructured biomimetic and bioresorbable implant with a porous composite structure, mimicking the whole osteochondral anatomy with its three different layers:

- superficial layer (100% type I collagen): smooth surface, reproducing the articular surface.
- intermediate layer (60% type I collagen and 40% hydroxyapatite): tidemark-like layer.
- lower layer (30% type I collagen and 70% hydroxyapatite): reproducing the composition of the subchondral bone [13].

Currently in the literature, there is still no evidence regarding the use of MaioRegen in the treatment of osteochondral knee defects, despite numerous articles reporting its use; in particular, the literature is scarce regarding the randomized clinical trials.

This systematic review aims to investigate the clinical and radiological efficacy of MaioRegen in restoring osteochondral knee defects.

## 2. Experimental Section

### 2.1. Data Search Protocol

**Figure 1:**
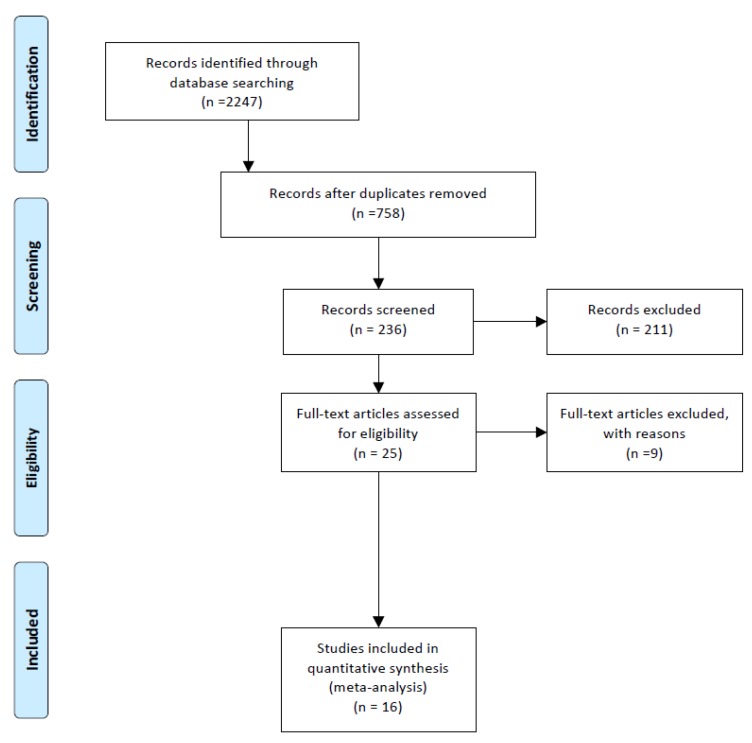
A flowchart of the literature screening performed in this study.

A systematic review of the existing literature was performed to identify all studies dealing with MaioRegen scaffold for the treatment of chondral and osteochondral knee defects. The Preferred Reporting Items for Systematic Reviews and Meta-analyses (PRISMA) guidelines were followed for the identification of the articles [14]. The research was performed by two independent investigators using MEDLINE, Scopus, CINAHL, Embase, and Cochrane Databases up to June 2018. Main search items were “MaioRegen”, “biomemetic scaffold”, “multilayered scaffold”, “scaffold”, “osteochondral lesion”, “cell-free scaffold”, “biocomposite scaffold”. The complete search strategy is shown in Figure 1. Additionally, reference lists of the selected articles were screened for further publications.

### 2.2. Study Selection and Eligibility Criteria

This systematic review includes studies of level I to IV (according to the “The Oxford 2011 Levels of Evidence” [15]).

The articles were analyzed regardless of their title and abstract by the two investigators. In case of disagreement, the papers were discussed until agreement was reached. In this systematic review, the inclusion criteria were: article that reported clinical, histological or radiological outcome data in patients treated with MaioRegen scaffold for osteochondral knee defect, article written in English, study of level I, II, III or IV; article published between January 1990 and June 2018.

Exclusion criteria were: article not written in English, case series with less than 5 cases, case reports, editorials, systematic reviews, and meta-analysis.

### 2.3. Data Extraction, Synthesis, and Analysis

Three independent reviewers analyzed all the information available from the articles (data and journal of publication, type of the study level of evidence, demographics data, diagnosis, surgical procedure, follow-up duration, outcomes, and complications) and entered it into a spreadsheet for analysis. Inter-observer agreement, was 0.95 (PRISMA guidelines) [16].

Results from the early postoperative period (12 months), intermediate postoperative period (12–24 months), and long-term follow-up results (>24 months) were gathered. Radiological information about defect filling, integration of newly formed cartilage with the adjacent cartilage, the cartilage surface quality, and the properties of the subchondral bone was extracted. Moreover, the rate of failure meant as the need for successive knee arthroplasty or realignment surgery was recorded. Complications, such as adverse reactions, infections, or patients who underwent a second surgery (including knee mobilization) were collected too. Concomitant surgeries were not considered in the study.

## 3. Results

The initial search resulted in 2,247 articles, of which 16 articles were selected based on the eligibility criteria (Figure 1). Patient characteristics of the included studies are shown in Table 1 [17,18,19,20,21,22,23,24,25,26,27,28,29,30,31,32]. Of the 16 studies, 15 [17,18,19,20,21,23,24,25,26,27,28,29,30,31,32] (93.75%) were case series (Level IV) and only one [22] (6.25%) a comparative study (Level III). All articles were published between 2011 and 2018. Mean number of patients for the study was 29.44 ± 17.66.

### 3.1. Demographic Results

Data from a total number of 471 patients were included in this systematic review. The mean age was 34.07 ± 5.28 years. The treatment involved 500 lesions divided as follows: 202 (40.4%) medial femoral condyles, 107 (21.4%) lateral femoral condyles, 28 (5.6%) tibial plateaus, 46 (9.2%) trochleas, 74 (14.8%) patellas, and 43 (8.6%) unspecified femoral condyles. Mean superficial lesion size was 3.6 ± 0.85 cm^2^. All lesions were classified as Grade III or IV (ICRS classification), except for two studies in which patients were treated for spontaneous osteonecrosis of the knee (SPONK) or unicompartmental knee osteoarthritis grade 3 (Kellgren–Lawrence classification). Only four studies [23,26,29,31] (25%) reported a follow-up longer than 24 months and only two studies [23,27] (12.5%) reported a post-operative histological evaluation. Almost all the articles reported clinical data using Tegner score (15/16–93.75%) [15,17,18,19,20,21,22,23,24,25,26,27,28,29,30] or International Knee Documentation Committee score (IKDC) (14/16–87.5%) [15,16,17,18,19,20,21,22,23,24,25,26,27,29]. Other reported clinical scores were Knee Injury and Osteoarthritis Outcome Score (KOOS), EuroQol-visual analog scales (EQ-VAS), Cincinnati, and Lysholm [17,18,19,30,32]. Tegner activity level scale is a graduated list of activities of daily living, recreation, and competitive sports. The patient is asked to select the level of participation that best describes their current level of activity and that before injury. The score varies from 0–10. A score of 0 represents sick leave or disability pension, whereas a score of 10 corresponds to participation in national and international elite competitive sports. The International Knee Documentation Committee is a knee-specific patient-reported outcome measure. The IKDC Questionnaire is a subjective scale that provides patients with an overall function score. The questionnaire looks at 3 categories: symptoms, sports activity, and knee function. Scores are obtained by summing the individual items, then transforming the crude total to a scaled number that ranges from 0 to 100. The Cincinnati knee-rating system has 11 components including sections that measure physical examination, instrumented knee stability, testing, and radiographic findings. Lysholm consists of 8 different items on a 100-point scale with 25 points each attributed to instability and pain. The EQ VAS records the patient’s self-rated health on a vertical visual analog scale. This can be used as a quantitative measure of health outcome that reflects the patient’s own judgment.

### 3.2. Clinical Outcome in Early Postoperative Period (12 Months)

Thirteen studies (81.25%) [17,19,20,21,22,23,24,25,27,28,30,31,32] showed a clinical significant improvement (IKDC, Lysholm and KOOS) at 12 months follow-up in comparison with the pre-operative value (*p* < 0.05). Only one study (6.25%) reported no clinical significant improvement [18] (*p* > 0.05). A significant improvement (*p* < 0.05) was also reported comparing data at 6 and 12 months follow up (measured by IKDC) in one study (6.25%) [20]. Eleven studies (68.75%) [19,20,21,22,24,25,27,28,30,31,32] collected Tegner score data at one year after surgery, of which only seven (43.75%) [20,21,22,24,25,27,28] showed a significant improvement (*p* < 0.05), while four studies (25%) [19,30,31,32] showed similar or inferior score at follow-up with respect to pre-operative value.

### 3.3. Clinical Outcome at Intermediate Follow-Up (24 Months)

Two years after surgery, thirteen (81.25%) [17,19,20,21,22,23,24,25,26,27,28,31,32] studies reported a clinical (IKDC, Lysholm, and KOOS) significant improvement compared with pre-operative values (*p* < 0.05). Likewise, early follow-up, the study of Brix et al. reported no significant clinical improvement [18]. Furthermore, the most interesting data are found in seven studies (43.75%) [17,22,23,24,25,27,28]; a clinically significant improvement (IKDC) was observed between 12 and 24 months (*p* < 0.05). For what concerns sports activity, eleven studies (68.75%) [17,20,22,23,24,25,26,27,28,31,32] reported a significant clinical improvement of Tegner Score compared with pre-operative values (*p* < 0.05); in two studies (12.5%) that also increased significantly between 12 and 24 months [25,28] (*p* < 0.05).

### 3.4. Clinical Outcome in Long Term Follow-Up (>24 Months)

Only four studies (25%) [23,26,29,31] reported clinical follow-up longer than 24 months. All of them showed a significant improvement in IKDC and Tegner scores versus pre-operative value (*p* < 0.05), with stable results with respect to the previous follow-up. Only one study (6.25%) [31] reported a significant improvement in IKDC score at 60 months after surgery with respect to 24-month follow-up (*p* < 0.05).

### 3.5. Radiological Evaluation

In four studies [19,20,22,29] (25%) radiological results were not reported. Eleven studies (68.75%) [17,18,21,23,24,25,26,27,28,31,32] evaluated the treatment using magnetic resonance observation of cartilage repair tissue (MOCART) score. The MOCART score was designed to evaluate the treatment of chondral and osteochondral lesions in as subjective a way as possible. This scale evaluates the appearance of the repaired tissue, the covering of the lesion, the integration of the margins, the intensity of the signal, and the state of the subchondral lamina. The score ranges from a minimum of 0 (worst possible result) to a maximum of 100 (best possible result).

In one study (6.25%) [30], the authors performed an evaluation with single photon emission computed tomography/computed tomography (SPECT/CT).

Moreover, in one study (6.25%) [18] T2 mapping was used to evaluate the cartilage quality. In three studies (18.75%) [26,28,32], a significant improvement among consecutive follow-ups in MOCART score was reported (*p* < 0.05). The SPECT/CT performed at 12 months [30], reported a complete filling of the defect in 14%, a partial filling in 14%, and only minor filling in 72% of patients, while at 18 months the T2 mapping showed a mean zonal T2 index in the repair tissue of 0.9874 which differed significantly compared to the healthy control cartilage [18] (*p* < 0.05).

### 3.6. Histological Evaluation

Only two studies (12.5%) [23,27] reported a histological evaluation. In the first study, Berruto et al. showed in all the specimens no residual scaffold [23]. Both subchondral bone and the mineralization process appeared normal in all the specimens evaluated with Mallory trichrome staining; Kon et al. reported complete biomaterial reabsorption and a hyaline-like tissue with a strong proteoglycan content and presence of collagen type II at 2-year follow-up [27].

### 3.7. Minor Complications

A total of 52 (11.1%) minor complications were reported, of which 16 experienced joint stiffness, 5 fever, and 31 swelling and bleeding of the knee.

### 3.8. Major Complications

A total of 7 (1.48%) major complications were reported of which 2 arthroscopic regularization/shaving due to partial detachment of the scaffold, 1 arthroscopy due to a novel chondral lesion on the lateral condyle, and 1 arthroscopy to remove an infrapatellar ossicle, 1arthroscopy for graft loosening, and 2 graft hypertrophy.

### 3.9. Failures

A total of 16 (3.39%) failures were reported.

## 4. Discussion

This systematic review aimed to evaluate existing literature concerning the use of MaioRegen for the treatment of osteochondral knee defects in humans, analyzing all relevant medical databases. The main finding of this systematic review is that MaioRegen can be considered a safe alternative treatment for an osteochondral defect with a low rate of complications or failures.

MaioRegen is a tri-phasic scaffold incorporating a biomimetic design that attempts to resemble the structure of osteochondral tissue as closely as possible [11,13]. Using the nucleation of HA nanocrystals onto self-assembled collagen fibers, this biomaterial was generated so that it could mimic the hierarchical layered structure of osteochondral tissue while also resembling the composition of the extracellular matrix’s (ECM) of cartilage and bone tissues [11,13].

The positive clinical results reported in this review highlight the advantages of having a hierarchical graded structure that mimics more closely the natural structure of the osteochondral tissue [10,11].

Histological results emphasize that no residual scaffold was identified with a complete resorption of the graft suggesting that the scaffold was progressively replaced and substituted by new tissue.

The studies included in this review demonstrated that MaioRegen is effective and reliable over time; in fact, almost all the studies reported a significant improvement in the first year after surgery, and seven studies (43.75%) [17,22,23,24,25,27,28] reported further improvement between 12 and 24 months. Only one study (6.25%) also reported a significant clinical improvement at 60 months of follow-up if compared with clinical results at 24 months [31] showing the durability of the outcomes.

A recent systematic review highlighted an increasing number of articles dealing with cell-free scaffolds both in clinical and preclinical studies [33]. This is not surprising because in recent years these scaffolds have gained more and more popularity and huge steps forward were made regarding biomaterials research with the development of a new generation of materials able to mimic the characteristics of human tissues and exploit the intrinsic tissue regeneration ability and avoid the risk linked to bacterial contamination and phenotype loss during extensive cell manipulation; therefore, reducing costs and simplifying the procedure. In this way, scaffolds that aim to restore osteochondral lesions were developed, with the challenge to guide the regeneration of 2 different tissues characterized by different healing potentials, and promising results have been shown in the clinical setting as well for the treatment of complex cases. This technique is robust and easy for surgeons to handle, and was reported to improve the healing of cartilage defects significantly [33].

On the other hand, there are still several problems associated with the safety and long-term effectiveness of these materials. Synthetic polymers can have potential problems of retention and degradation in situ. Biological materials potentially carry the risk of transmitting infectious agents and starting immunological reactions. Currently, in the literature, there are no studies comparing scaffold-based and scaffold-free approaches for the treatment of osteochondral defects and is still unknown which could be considered the gold standard. A scaffold-free cell delivery system can be considered an excellent alternative due to its simplicity both for development and implantation [33,34].

To date, in the literature, clinical results are available only regarding the use of other two synthetic osteochondral scaffolds: TruFit (Smith & Nephew, Andover, MA, USA) and Agili-C (CartiHeal, 2009-Ltd, Kfar Sava, Israel) [35,36].

The TruFit plug is a synthetic, acellular scaffold and is predominantly made from a polylactide-coglycolide copolymer. The scaffold consists of two “phases.” The bone phase contains calcium sulfate for stimulation of bone formation. Cartilage regeneration is instigated by the integration of cells and growth factors derived from the bone marrow that infiltrates the plug [37].

A systematic review published in 2015 reported clinical improvement at 12-months follow-up compared to pre-operatively using the TruFit plug; however, two studies reporting longer follow-up showed a deterioration of early improvement [38]. Radiological evaluation indicates favorable MRI findings regarding filling of the defect and incorporation with adjacent cartilage at 24 months follow-up using TruFit, but conflicting evidence exists on the properties of the newly formed overlying cartilage surface. None of the included studies showed evidence for bone ingrowth. The minimal histological data available confirmed these results [38].

Agili-C, an aragonite-base osteochondral scaffold, consists of two layers: (1) the bone phase represented by calcium carbonate in the aragonite crystalline form, and (2) the superficial cartilage phase composed by modified aragonite and biodegradable and biocompatible hyaluronic acid [36]. Preclinical analysis revealed the safety and potential of this scaffold, showing its biodegradability and intrinsic restorative potential. In particular, the scaffold was able to recruit cells from the surrounding tissue which allowed good regeneration of the entire osteochondral unit. In fact, it was demonstrated that chondro-progenitor cells with migratory ability are also present in pathological cartilage tissue. Their colony forming ability and their paracrine activity characterized by the release of chondrogenic, angiogenic and pro-mitogenic molecules [39] set the appropriate conditions to promote the scaffold integration. Therefore, the scaffold can be translated into the clinical setting as a technique for one-step implantation also without cell augmentation [36]. In a recent clinical study, 21 patients without severe osteoarthritis received tapered shaped aragonite-based scaffolds for the treatment of 2.5 ± 1.7 cm^2^ knee cartilage defects. The control group consisted of 76 patients selected according to the same criteria from a database of patients who previously underwent implantation of cylindrical-shaped implants. A statistically significant improvement in all clinical scores was reported both in the tapered and cylindrical group. No difference could be detected between the improvement obtained with the two implant types, neither in the clinical nor in imaging evaluations. A difference could be detected, instead, in terms of the revision rate, which was lower in the tapered implant group with no implant removal—0% vs. 8/76–10.5% failures in the cylindrical implants [40]. This can be reflective of a better preservation of the scaffold integrity during insertion, with the maintenance of a better press-fit and consequently an enhanced incorporation and in the end an improvement in terms of adverse events and failures while offering the same benefits of the more widely documented cylindrical implants in terms of clinical outcome [40].

Among the advantages of using synthetic scaffold is the lack of donor site morbidity. In fact, a systematic review analyzing the knee donor-site morbidity after mosaicplasty showed that the donor-site morbidity for knee-to-ankle (16.9%) was greater than knee-to-knee (5.9%) mosaicplasty procedures without any significant correlation between the rate of donor-site morbidity and size of the defect, and number and size of the plugs [41].

Another important finding in our review is the return to sport in patients who underwent MaioRegen treatment. After 24 months from surgery, eleven (68.75%) [17,20,22,23,24,25,26,27,28,31,32] studies reported a clinical improvement in Tegner score, with further improvement in two studies (12.5%) at 24 months [25,28]. Literature regarding return to sport using osteochondral scaffolds such as TruFit or Agili-C is scarce but a systematic review published in 2016 compared return-to-sport outcomes after microfracture (MFX), osteochondral autograft transfer, osteochondral allograft transplantation, and autologous chondrocyte implantation at a minimum follow-up of 2 years [42]. Data about 2549 athletes who had undergone cartilage restoration surgery showed that 76% returned to sport at mid-term follow-up. Osteochondral autograft transfer offered a faster recovery and appeared to have a higher rate of return to preinjury athletics but heterogeneity in lesion size, athlete age, and concomitant surgical procedures are important factors to consider when assessing individual athletes [42].

Our systematic review presents several limitations: first of all studies are level IV and only one is level III, highlighting the lack of randomized trials in this field and therefore limiting the quality and reliability of these findings.

Another limitation of our study is the fact that we have not considered the additional procedures that have been performed in patients; the purpose of the study was to analyze exclusively the use of the MaioRegen and considering the other surgical times would have required a stratification of the patients, creating possible bias, particularly in such a small cohorts of patients.

## 5. Conclusions

This systematic review describes the currently available evidence for the treatment of osteochondral knee defects with MaioRegen Osteochondral substitute reporting promising satisfactory and reliable results at mid-term follow-up while considering the return to sport. A low rate of complications and failure was reported, therefore, confirming the safety of this scaffold. Considering the low level of evidence of the study included in the review, this data does not support the superiority of the Maioregen in terms of clinical improvement at follow-up compared to conservative treatment or other cartilage techniques. Many doubts still exist regarding the ability to regenerate both hyaline cartilage formation and subchondral bone ingrowth, well-designed, large-scale, randomized controlled trials are needed to investigate the value of future synthetic scaffolds.

## Figures and Tables

**Table 1:** Clinical studies investigating the use of MaioRegen for osteochondral knee defects.

Article	Type of Study	Number of Patients/Mean Age	Location	Lesion Size	Follow-Up	Clinical Results		Radiologic Results/Histological Evaluation	Complications/Reoperation
						Preop	Postop	Postop	
Delcogliano et al. 2014, KSSTA [17]	Case Series, Level IV	19 patients; 33 ± 15 years	20 lesions: 10 MFC, 7 LFC, 3 tibial plateau	5.2 ±1.6 cm^2^. (4–8 cm^2^); mean depth of 8 mm (6–9 mm)	12 and 24 months	IKDC: 35.7 ± 6.3 Tegner: 2 (0–4) EQ-VAS: 3.1 ± 1.1	IKDC: 12 months: 67.7 ± 13.4 (*p* < 0.0005) * 24 months: 72.8 ± 12.4 (*p* < 0.0005) *^†^ Tegner: 24 months: 5 (1–7) (*p* < 0.0005) * EQ-VAS 24 months: 7.3 ± 1.1(*p* < 0.0005) *	MOCART 24 months: 63.2 ± 11.7	2 failures: 12 and 24 months after surgery No complications
Brix et al. 2016, Int Orthop [18]	Case Series, Level IV	8 patients; 37 years	8 lesions: 5 MFC, 3 LFC	2.07 cm^2^ (1.5 to 3.75 cm^2^)	12,18 and 24 months	IKDC Lysholm Cincinnati	IKDC: 6,12,18 and 24 months: *p* = 0.30 Tegner–Lysholm: 6,12,18 and 24 months: *p* = 0.176 Cincinnati 6,12,18 and 24 months: *p* = 0.53	MOCART 18 months: 69 (60–100) T2 mapping at 18 months: 0.9874	2 complications: resurgery at 7 and 24 months not for scaffold failure. In the first case the re-arthroscopy showed a novel chondral lesion on the lateral condyle. In the second case an infrapatellar ossicle was removed No failures
Berruto et al. 2016, The Knee [19]	Case Series, Level IV	11 patients; 52.1± 9.6 years	11 lesions: 9 MFC, 2 LFC	3.47 ± 1.75 cm^2^ (range 1.5 to 7.5 cm^2^)	12 and 24 months	IKDC: 40.54 ± 15.0 (17–57) Lysholm: 49.7 ± 17.9 (22–88) Tegner: 4 ± 1.1 (2–6)(pre-injury) VAS: 6.3 ± 2.5 (3–8)	IKDC: 12 months: 65.72 ± 14.8 * (*p* = 0.014) 24 months: 63.90 ± 19.9 * (*p* = 0.03) Lysholm: 12 months: 85.4 ± 12.1 * (*p* = 0.01) 24 months: 86.6 ± 12.7 * (*p* = 0.04) Tegner: 12 months: 3.7 ± 1.3 ( *p* > 0.05) 24 months: 3.8 ± 1.7 ( *p* > 0.05) VAS: 12 months: 2.2 ± 2.1 * (*p* = 0.02) 24 months: 1.6 ± 2.7 * (*p* < 0.05)	N.A.	2 failures: 2 condylar collapse and subsequent knee arthroplasty at 18 months after implantation
Kon et al. 2014, Injury [20]	Case Series, Level IV	11 patients; 37.3 ± 11.0 years	13 lesions: 11 tibial plateau, 1 MFC, 1 LFC	5.1 ±2.7 cm^2^ (3.0–12.5 cm^2^)	6,12 and 24 months	IKDC: 42.5 ± 10.2 Tegner: 2.3 ± 2.1	IKDC: 6 months: 58.3 ± 14.1 * (*p* < 0.05) 12 months: 69.8 ± 19.0 *^†^ (*p* = 0.03) 24 months: 68.4 ± 17.0 * Tegner: 12 months 4.8 ± 2.4 * (*p* < 0.05) 24 months: 5.3 ± 2.5 *	N.A.	3 minor complications: fever during the first week spontaneously resolved.
Di Martino et al. 2015, Injury [21]	Case Series, Level IV	23 patients; 38.0 ±8.2 years	23 lesions: 12 MFC, 9 LFC, 1 tibial plateau, 1 patella	3.2 ± 1.9 cm^2^	12 and 24 months	IKDC: 42.8 ± 13.8 Tegner score: 3.3 ± 2.7 (before injury 6.1 ± 2.6)	IKDC: 12 months: 74.3 ± 17.4 *(*p* < 0.0005) 24 months 74.9 ± 2 0.4 * Tegner: 12 months: 4.6 ± 2.2 * (*p* < 0.0005) 24 months: 4.7 ± 2.1 *	Mocart score: 12 monhts: 72.9 ± 13.6 24 months: 70.8 ± 13.2	2 complications: 2 patients underwent knee mobilization under narcosis at 2 and 4 months 2 failures
Filardo et al. 2013, The Knee [22]	Comparative study, Level III	33 patients, 39.5 ± 10.6 years	47 lesions: 11 MFC, 9 LFC, 13 trochlea, 9 patella, 5 tibial plateau	4.5 ± 2.7 cm^2^	12 and 24 months	IKDC: 40.4 ± 14.1 VAS: 4.0 ± 2.2 Tegner: 1.9 ± 1.8	IKDC: 12 months: 69.6 ± 17.0 * (*p* < 0.0005) 24 months: 75.5 ± 15.0 *^†^ (*p* = 0.038) VAS: 12 months: 4.7 ± 3.0 24 months: 7.3 ± 1.5 *^†^ (*p* < 0.0005) Tegner: 12 months: 4.0 ± 1.8 * (*p* < 0.0005) 24 months: 4.5 ± 1.7 * (*p* = 0.09)	N.A.	No failure 2 complications: 2 arthroscopic regularization/shaving due to partial detachment of the scaffold
Berruto et al. 2014, Am J Sports Med [23]	Case Series, Level IV	49 patients; 37.6 ± 14 years	49 lesions: 33 MFC, 11 LFC, 4 tibial plateau, 1 trochlea	4.35 cm^2^ (3–8.25 cm^2^)	6,12 and 36 months	IKDC: 45.45 ± 19.29 VAS: 6.69 ± 1.88 Tegner: 2.20 ± 0.67	IKDC: 12 months: 70.86 ± 18.08 * (*p* < 0.001) 24 months: 75.42 ± 19.31 *^†^(*p* < 0.05) 36 months: 76.14 ± 18.53 * VAS: 12 months: 2.55 ± 2.38 * (*p* < 0.05) 24 months: 1.96 ± 2.47 * 36 months: 2.1 ± 2.23 * Tegner score: 24 months: 4.9 ± 1.73 * 36 months: 5.06 ± 1.65 *	MOCART 24 months: 70% showed complete filling of the lesion, 63.3% had an intact articular surface, and 86% had mild or no effusion Histological evaluation: In all specimens, histological examination revealed no residual scaffold. Both subchondral bone and the mineralization process appeared normal in all specimens evaluated with Mallory trichrome	6 minor complications (swelling and bleeding) 5 failures
Perdisa et al. 2017, Am J Sports Med [24]	Case Series, Level IV	34 patients; 30.0 ± 10 years	34 patellar lesion	2.1 ± 1 cm^2^	12 and 24 months	IKDC: 39.5 ± 14.5 Tegner: 1.8 ± 1.0	IKDC: 12 months: 61.9 ±14.5 * (*p* < 0.0005) 24 months: 67.6 ± 17.4 *^†^ (*p* = 0.02) Tegner: 12 months: 3.3 ± 1.5 * (*p* < 0.0005) 24 months:3.3 ± 1.1 *	MOCART: 12 months: 79.8 ± 12.6 24 months: 83.5 ± 11.4	2 failures, underwent realignment procedures
Filardo et al., 2013, Am J Sports Med [25]	Case Series, Level IV	27 patients; 25.5 ± 7.7 years	27 lesions: 17 MFC, 10 LFC	3.4 ± 2.2cm^2^	12 and 24 months	IKDC: 48.4 ± 17.8 Tegner: 2.4 ± 1.7 (5.7 ± 2.3 before onset of symptoms)	IKDC: 12 months: 76.0 ±12.8 * (*p* < 0.0005) 24 months: 82.3 ± 12.8 *^†^ (*p* < 0.0005) Tegner: 12 months:3.6 ± 1.2 * (*p* = 0.01) 24 months:4.5 ± 1.6 *^†^ (*p* = 0.01)	MOCART: 12 months: 66.9 ± 12.8 24 months: 67.0 ± 25.7	5 adverse event: -3 patients had joint stiffness treated with knee mobilization under anesthesia -2 fever
Kon et al. 2014, Am J Sports Med [26]	Case Series, Level IV	27 patients; 34.9 ± 10.2 years	30 lesions: 7 MFC, 5 LFC, 11 patella, 5 trochlea 2 tibial plateau	2.9 ± 1.3 cm^2^	24 and 60 months	IKDC: 40.0 ± 15.0 Tegner:1.6 ± 1.1 (5.2 ± 2.6 before onset of symptoms)	IKDC: 24 months: 76.5 ±14. 5 * (*p* < 0.0005) 60 months: 77.1 ±18.0 * (*p* < 0.000) Tegner: 24 months: 4.0 ± 1.8 * (*p* < 0.0005) 60 months:4.1 ± 1.9 * (*p* < 0.0005)	MOCART: 24 months:68.0 ± 13.8 60 months: 74.8 ± 12.3 ^†^	None
Kon et al. 2011, Am J Sports Med [27]	Case Series, Level IV	28 patients; 35.3 ± 10.2 years	34 lesions: 8 MFC, 5 LFC, 12 patella, 7 trochlea, 2 tibial plateau	2.9 ± 1.3 cm^2^	6,12 and 24 months	IKDC Tegner:1.6 ± 1.1 (5.2 ± 2.5 before onset of symptoms)	IKDC: 6 months: *p* < 0.0005 * 12 months: *p* < 0.0005 * 24 months: *p* < 0.005 *^†^ Tegner: 12 months:4.0 ± 1.6 * (*p* < 0.0005) 24 months: 4.0 ± 1.6 * (*p* < 0.0005)	MOCART: 24 months: 79.2 (40–95) Histological evaluation: at the 2-year follow-up showed complete biomaterial reabsorption and a hyaline-like tissue with a strong proteoglycan content and presence of collagen type II	10 complications: Swelling during the first month was observed in 6 patients. One patient experienced bleeding during the first 3 days after surgery. Two patients developed a fever during the first 3 weeks. All adverse events resolved within 1 month after surgery, with the exception of 2 patients with joint stiffness who were reoperated on arthroscopically, one at 2 months and the other at 5 months. One patient affected by multiple lesions had loosening of one of the grafts, which was removed, and another patient was reoperated for graft hypertrophy.
Kon et al. 2014, J Mater Sci: Mater Med [28]	Case Series, level IV	79 patients; 31.0 ± 11.3 years	82 lesions: 41 MFC, 26 LFC, 15 trochlea	3.2 ± 2.0 cm^2^	12 and 24 months	IKDC: 47.4 ± 17.1 Tegner: 2.9 ± 2.0 (6.3 ± 2.2 before the onset of symptoms)	IKDC: 12 months: 72.1 ±18. 9 * (*p* < 0.0005) 24 months: 76.2 ±19.6 *†(*p* = 0.004) Tegner: 12 months: 3.8 ± 1.6 * (*p* < 0.0005) 24 months: 4.4 ± 1.9 *^†^ (*p* < 0.0005)	MOCART: 12 months; median 70 24 months: median 80†	17 patients reported swelling 9 resurgery due to stiffness
Marcacci et al. 2013, KSSTA [29]	Case Series, Level IV	43 patients; 40.1 ± 11	43 Femoral condyles	4.6 ± 2.1 cm^2^	36 months	IKDC: 47.3 ± 17.1 VAS: 6.1 ± 2.0 Tegner: 2 (1–5) 6 (3–10 before onset of symptoms)	IKDC: 79.6 ± 16.1 * (*p* < 0.0005) VAS: 2.3 ± 2.2 * (*p* < 0.0005) Tegner: 4 (3–10) * (*p* < 0.0005)	N.A.	None
Mathis et al. 2018, KSSTA [30]	Case Series, Level IV	14 patients; 33 ± 10 years	14 lesions: 8 MFC, 2 CFL. 2 trochlea, 2 patella.	1.0–3.5 cm^2^	12 months	Lysholm: 65.6 ± 12.6 Tegner: 6.0 (3-9)	Lysholm: 90.1 ± 10.0 * (*p* < 0.001) Tegner: 4.5 (*p* < 0.01) *	SPECT/CT: A complete filling of the defect was shown in 14%, a partial filling in 14% and only minor filling was seen in 72%.	None
Perdisa et al.2018, Am J Sport Med [31]	Case Series, Level IV	27 patients; 25.5± 7.7 years	27 lesions: 17 MFC, 10 LFC	3.4 ± 2.2cm^2^	12,24,36,48 and 60 months after surgery	IKDC: 48.4 ± 17.8 Tegner: 2.4 ± 1.7 5.7 ± 2.2 (before onset of symptoms)	IKDC: 12 months: *p* < 0.0005 vs preop * 24 months: *p* < 0.0005 vs preop * 36 months: *p* < 0.0005 vs preop * 48 months: *p* < 0.0005 vs preop * 60 months: *p* < 0.0005 vs preop and vs 24 months * Tegner: 12 months: non-significant improvement vs preop 24 months: 4.4 ± 1.6 (*p* = 0.001) * 60 months: 5.0 ± 1.7 (*p* < 0.0005)*	MOCART: 24 months: 74.2 ±16.2 60 months: 81.4 ±11.8	None
Verdonk et al., 2015, Bone Joint J [32]	Case Series, Level IV	38 patients; 30.5 ±11.9 years	38 lesions: 23 MFC, 7 LFC, 5 patella, 3 trochlea	3.7 ±2.4 cm^2^	3,6,12,18,24 months	KOOS: 213.9 ± 88.3 Tegner: 3.1 ± 2.5	KOOS: 3 months: 261.2 ± 98.8 * (*p* = 0.01) 6 months: 295.9 ± 106.1 * (*p* = 0.01) 12 months: 328.2 ± 105.7 * (*p* = 0.01) 18 months: 335.8 ± 100.9 * (*p* = 0.01) 24 months: 356.1 ± 96.9 *(*p* = 0.01) Tegner: 3 months: 1.9 ± 1.9 * (*p* = 0.01) 6 months: 2.5 ± 1.9 (*p* = 0.09) 12 months: 3.4 ± 1.9 (*p* = 0.46) 18 months: 3.4 ± 1.8 (*p* = 0.27) 24 months: 3.8 ± 1.9 * (*p* = 0.03)	MOCART: Significant improvement at 3, 12 and 24 months after surgery.	2 failures: total knee arthroplasty at 14 and 20 months 3 complications: -1 further arthroscopy due to hypertrophy -2 joint stiffness treated with knee mobilization under anesthesia

* Statistical significant difference vs. pre-op; † statistical significant difference vs. previous follow-up; MFC: Medial femoral condyle; LFC: Lateral femoral condyle; IKDC: International Knee Documentation Committee; EQ-VAS: EuroQol-visual analogue scales; VAS: Visual analogue scales; KOOS: Knee Injury and Osteoarthritis Outcome Score; MOCART: Magnetic resonance observation of cartilage repair tissue; SPECT/CT: Single photon emission computed tomography/Computed tomography.
